# Comparative transcriptomic analyses of thymocytes using 10x Genomics and Parse scRNA-seq technologies

**DOI:** 10.1186/s12864-024-10976-x

**Published:** 2024-11-11

**Authors:** Igor Filippov, Chinna Susan Philip, Leif Schauser, Pärt Peterson

**Affiliations:** 1 Molecular Pathology Research Group, Institute of Biomedicine and Translational Medicine, University of Tartu, Tartu, Estonia; 2 QIAGEN Aarhus A/S, Aarhus, Denmark

**Keywords:** Transcriptomics, 10x, Parse, scRNA-seq, Thymus

## Abstract

**Background:**

Single-cell RNA sequencing experiments commonly use 10x Genomics (10x) kits due to their high-throughput capacity and standardized protocols. Recently, Parse Biosciences (Parse) introduced an alternative technology that uses multiple in-situ barcoding rounds within standard 96-well plates. Parse enables the analysis of more cells from multiple samples in a single run without the need for additional reagents or specialized microfluidics equipment. To evaluate the performance of both platforms, we conducted a benchmark study using biological and technical replicates of mouse thymus as a complex immune tissue.

**Results:**

We found that Parse detected nearly twice the number of genes compared to 10x, with each platform detecting a distinct set of genes. The comparison of multiplexed samples generated from 10x and Parse techniques showed 10x data to have lower technical variability and more precise annotation of biological states in the thymus compared to Parse.

**Conclusion:**

Our results provide a comprehensive comparison of the suitability of both single-cell platforms for immunological studies.

**Supplementary Information:**

The online version contains supplementary material available at 10.1186/s12864-024-10976-x.

## Background

Single-cell RNA sequencing (scRNA-seq) has become an integral tool in immunology research as it enables studying the dynamics of various immune cell populations at high resolution [1]. In less than a decade, the exponential development in the field of scRNA-seq has streamlined multiple workflows with the possibility to analyze more than a million cells in the same kit with higher cell yields, higher sensitivity, straightforward and quicker workflows, and lesser instrumentation. These advancements in technology have promoted deeper understanding in numerous immune landscapes including thymic organogenesis and cellular development [2–4], cellular heterogeneity [5–7], ageing and diseased conditions [8–10], as well as the generation of large informative atlases [2, 11] in both mice and humans revealing potential clinical applications.

Among several scRNA-seq technologies, 10x Genomics (10x) is currently the de-facto leading platform providing a standardized way of processing individual cells with microfluidics benchtop equipment. 10x uses emulsion-based kits wherein individual cells are captured in water-in-oil emulsion droplets. 10x facilitates multi-omics studies, enabling simultaneous measurements of the immune receptors and cell surface proteins. Nevertheless, large-scale studies remain challenging due to the high reagent costs and substantial batch effects [12]. To overcome these challenges, experiments with multiple samples often use multiplexing techniques such as oligo-conjugated antibodies or lipids, which allow barcoding sample-specific cells [13].

Recently, Parse Biosciences developed an alternative scRNA-seq assay that relies on split-pool combinatorial indexing instead of microfluidics equipment [14]. Here, cells are fixed and permeabilized to facilitate the intracellular indexing. This is followed by four rounds of barcoding wherein transcripts are labelled with well-specific barcodes to ensure that each cell has a unique label consisting of a random combination of the four barcodes. Parse kits allow experiments with up to 96 samples without using molecular hashtags and enable scaling up to a million cells in a single run.

The wide use of scRNA-seq technologies has brought forth numerous challenges during cell processing as well as data analysis. Issues associated with cell processing include low cell numbers, low purity, and low target cell recovery which depends on various factors such as tissue type, cell type, dissociation method, and kit used. There are different approaches to analysis of scRNA-seq data and tricky issues have been raised at each stage from quality control to expression analysis irrespective of the approach. During quality control, one of the major challenges is dropouts wherein a gene is moderately or highly expressed in one cell but not detected in another cell [15]. However, this has been shown to be similar for both 10x and Parse [16]. Another challenge is background noise due to the presence of ambient RNA or barcode swapping [17]. Ambient RNA is majorly present in droplet-based technology and can be described as the mRNA released in cell suspension from damaged or dying cells which contaminates the true cellular gene transcripts [18]. Barcode swapping, on the other hand, is predominant in plate-based technology and involves mislabeling of gene transcripts with free floating barcodes [19]. Ambient RNA removal is essential to ensure accurate identification of cell types and to avoid biological misinterpretation of scRNA-seq data.

Several studies have recently comprehensively compared multiple scRNA-seq technologies including 10x and Parse kits in terms of cell processing and data analysis [16, 20–22]. However, most of these studies use human PBMCs or cell lines for comparison between technologies. Additional challenges to processing and sequencing single-cell transcriptomes are posed while working with immunological organs because of their cellular complexity and several differentiation and activation stages of the cells. One of these complex organs is the thymus, where T cells differentiate, starting from the double-negative CD4^−^CD8^−^ stage and progressing through double-positive CD4^+^CD8^+^ towards the single-positive CD4^+^ or CD8^+^ stage [23, 24]. Thymocytes are sensitive to apoptotic cell signals, and a large number of cells that fail the negative and positive selection are removed through apoptosis during normal differentiation [25]. Therefore, processing and analyzing scRNA-seq data requires vigilance to preserve and identify fragile populations.

In this study, we investigated the performance of 10x and Parse scRNA-seq methods on the thymus as an immune organ with a well-established cellular composition. We applied these methods to the thymus isolated from two age- and sex-matched C57BL/6N mice to assess their performance for cell processing as well as downstream data analysis by analyzing their ability to capture relevant cell states at a transcriptomic level. In addition, we included technical replicates to measure the degree of reproducibility of the single-cell experiments. To our knowledge, we present the first comprehensive benchmark evaluation of multiplexed commercial scRNA-seq solutions in a primary immune organ.

## Results

### Experiment design and data generation

Many studies have explored the heterogeneity of thymic populations using single-cell technology and provided a comprehensive outline of the distinct cell types and marker genes by sorting out specific thymocyte populations [11, 26–28]. We, therefore, used mouse thymus as a model tissue to study the performance of the 10x and Parse platforms and compare their quality control metrics in capturing single-cell transcriptome.

**Fig. 1:**
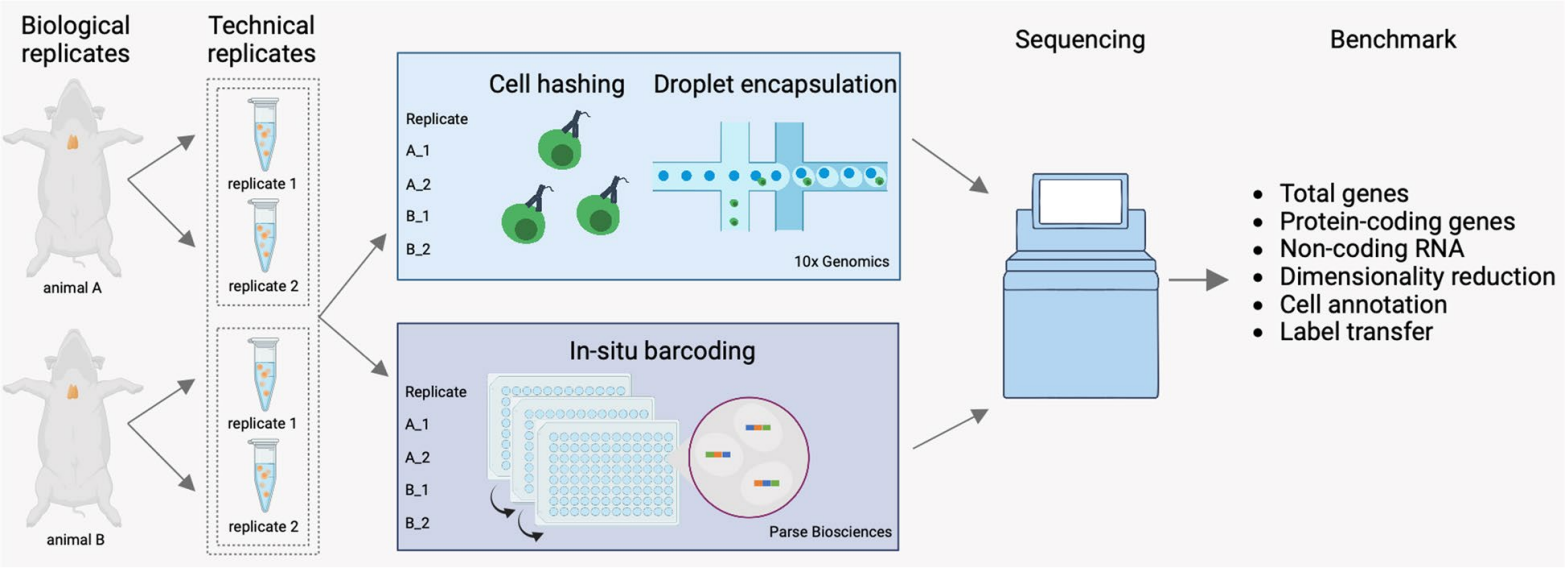
The experiment design. Thymi from two mice were divided into two technical replicates each. The resulting four samples were further divided between 10x and Parse workflows

**Fig. 2:**
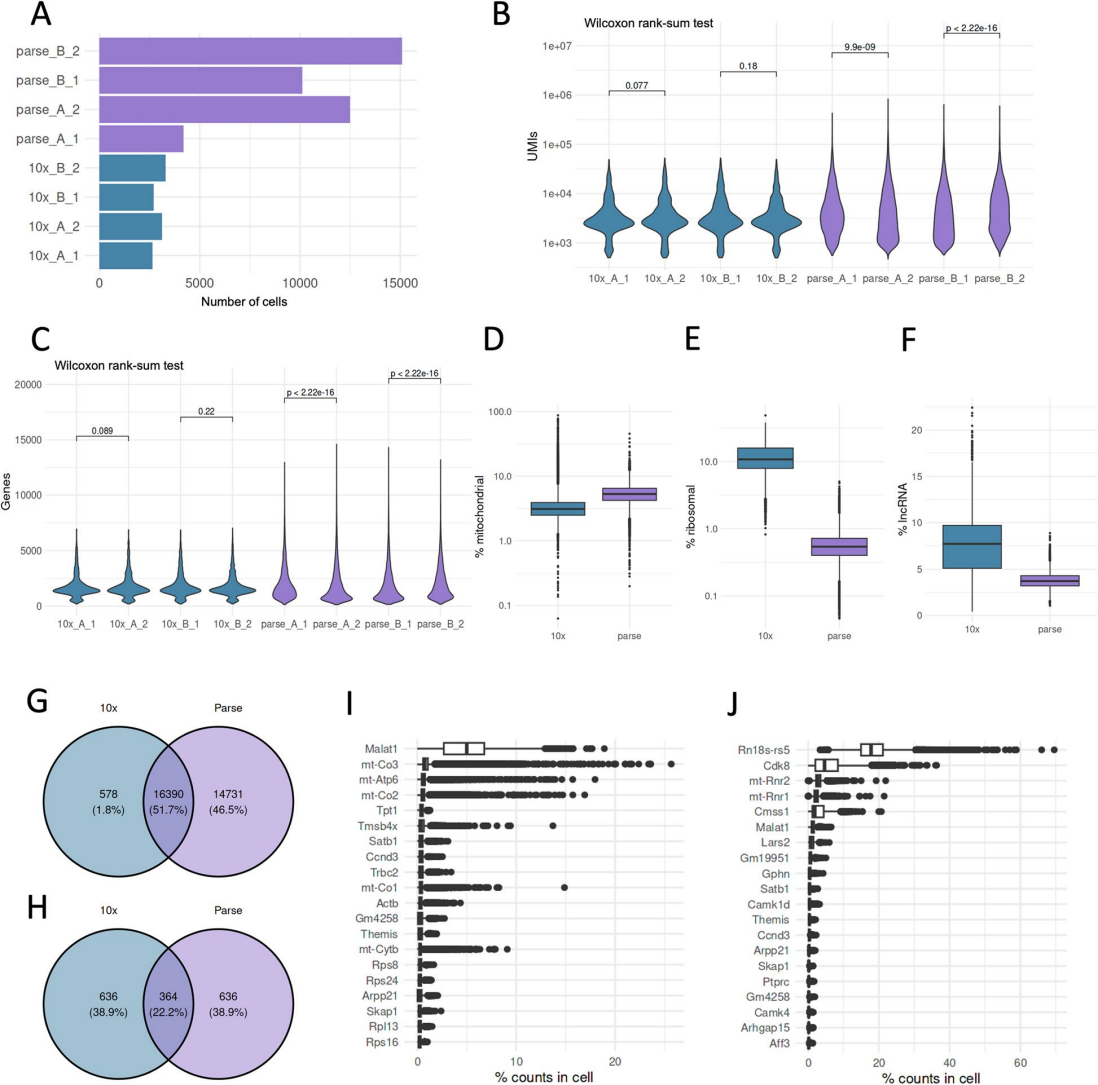
Cell recovery and gene detection. **A** Number of cells recovered from each replicate, (**B**) UMI counts/cell for each sample, (**C**) Genes detected/cell in each sample, (**D**) Percentage of expression mapped to mitochondrial genes, (**E**) Percentage of expression mapped to ribosomal genes, (**F**) Percentage of expression mapped to long non-coding RNA genes, (**G**) Top expressed genes in 10x library, (**H**) Top expressed genes in Parse library, (**I**) Overlap of all genes detected in both libraries, (**J**) Overlap of top 1000 expressed genes in both libraries

To this end, we used thymi isolated from two female C57BL/6N mice aged two months. The thymic lobes from each mouse (biological replicates A and B) were separated and considered as two technical replicates (replicate 1 and 2). After enzymatic digestion, the four thymic samples were divided and processed separately by 10x and Parse kits (Fig. 1). Hereafter, we refer to the 10x samples as 10x_A_1, 10x_A_2, 10x_B_1, and 10x_B_2. Similarly, Parse samples were designated as parse_A_1, parse_A_2, parse_B_1, and parse_B_2. The 10x samples were further labelled using cell hashing and subsequently processed using the 10x microfluidics device. The Parse samples were first processed with the cell fixation kit and the cells barcodes were added using the split-pool barcoding. We aimed to collect ∼3,000 cells for 10x per sample and 5,000 cells for Parse. Accordingly, we loaded ~ 5,100 cells per sample for 10x and ~ 19,200 cells for Parse. After sequencing, we recovered on average 3,477 cells per sample for 10x with negligible inter-sample variability and a recovery rate of 56.5% (Fig. 2A, Tables 1, S1, S2). Interestingly, we recovered on average 10,460 cells per Parse sample with high inter-sample variability and a recovery rate of 54.4% compared to the expected ~ 5,000 cells per sample (Fig. 2A, Tables 1, S1, S2). Thus, 10x showed higher cell capture efficiency and lower inter-sample variability.

**Table 1:** Sequencing statistics for 10x and Parse libraries

	Parse	10x
Cell recovery rate	54.4%	56.5%
Mean reads/cell	42,299	45,959
Sequencing saturation	71.2%	83.8%
Median UMIs/cell	3,128	2,998
Median genes/cell	1,355	1,570
cDNA > Q30	90.8%	91%
Total genes detected	31,121	16,968

### 10x and Parse show disparities in quality control measures

After demultiplexing the 10x data (Figure S1A), we examined the distributions of UMI and detected gene counts in each cell, as these are often used as quality control metrics in scRNA-seq data analysis. We analyzed the gene expression matrices using the *CellRanger* and *split-pipe* pipelines for 10x and Parse data, respectively. The data from both assays contained a comparable number of UMIs and genes per cell. However, we noted that Parse UMI and gene distributions had longer tails in the violin plot compared to 10x data, which indicated more outlier cells with high gene expression counts (Fig. 2B, C). We further checked whether these metrics significantly differed between the technical replicates in each dataset. Interestingly, 10x technical replicates were not different in the number of UMIs and genes detected in cells, whereas Parse showed a significant difference in technical replicates.

In addition to UMI and gene counts, the proportion of reads mapping to mitochondrial and ribosomal genes serve as quality control metrics in scRNA-seq experiments. Furthermore, the presence of long non-coding RNA (lncRNA) transcripts is indicative of the cell sample quality. For example, the presence of long non-coding RNA (lncRNA) transcripts is indicative of the cell sample quality [29–32]. Therefore, we examined how these control measurements compared between the 10x and Parse datasets. We observed that the average percentage of reads mapped to mitochondrial genes was slightly lower in the 10x library compared to Parse (4.4% vs 5.5%) (Fig. 2D). Conversely, the average fraction of ribosomal gene counts was an order of magnitude higher in 10x data (12.5% vs 0.6%) (Fig. 2E). Additionally, the proportion of lncRNA counts was higher in the 10x samples than with the Parse kit (7.5% vs 3.8%) (Fig. 2F). These results show that both assays had comparable mitochondrial proportions with notable differences in the ribosomal and lncRNA genes.

### 10x and Parse data are enriched for distinct gene sets

We next analyzed the differences between the captured genes inthe datasets. We first removed genes detected in fewer than three cells and then examined the number of genes in 10x and Parse data. Despite the comparable depth in sequencing, the Parse dataset detected a higher number of unique genes. With 16,390 genes shared between the datasets, 14,731 genes were detected only in Parse data, compared to 578 in 10x (Fig. 2G). We investigated whether this difference was detectable in highly expressed genes. When we compared the overlap between the 1000 most expressed genes in both data, only 364 genes were shared (Fig. 2H).

We found that lncRNA, *Malat1*, was the top expressed gene in 10x data (Fig. 2I). In contrast, *Rn18s-rs5,* a ribosomal RNA, was the top gene in Parse data, while *Malat1* was the sixth most expressed gene (Fig. 2J). Several mitochondrial genes were expressed at high levels in both 10x and Parse data. However, these differed between 10x (*mt-Co3*, *mt-Atp6*, *mt-Co1*, and *mtCo2*) and Parse (*mt-Rnr1* and *mt-Rnr2*). Notably, 10x and Parse top expressed genes included the *Themis* gene, which is involved in positive and negative T cell selection.

### Computational analysis identifies more doublets in Parse data

Doublets or multiplets occur when two or more cells are encapsulated in a single droplet, leading to mixed or hybrid transcriptomic profiles. Including these aggregates in downstream analysis can incorrectly identify rare cell types, intermediate cell states, or specific transcriptomic signatures. Our study utilized cell hashing for the 10x samples, enabling us to differentiate doublets from single cells. Cells that were positive for more than one sample hashtag were identified as doublets and excluded from further analysis. The doublets constituted 14% of the 10x dataset (Figure S1A, Table S3). The Parse toolkit does not provide a way to distinguish doublets in the same manner as 10x since it does not use hashtags to multiplex samples. To address this, we employed the computational doublet detection method available in *scDblFinder*, which identifies doublets by comparing cells to computationally simulated doublet profiles [33]. Notably, doublets constituted 31% of the Parse WT library (Table S3).

### Parse data has a considerable batch effect

**Fig. 3:**
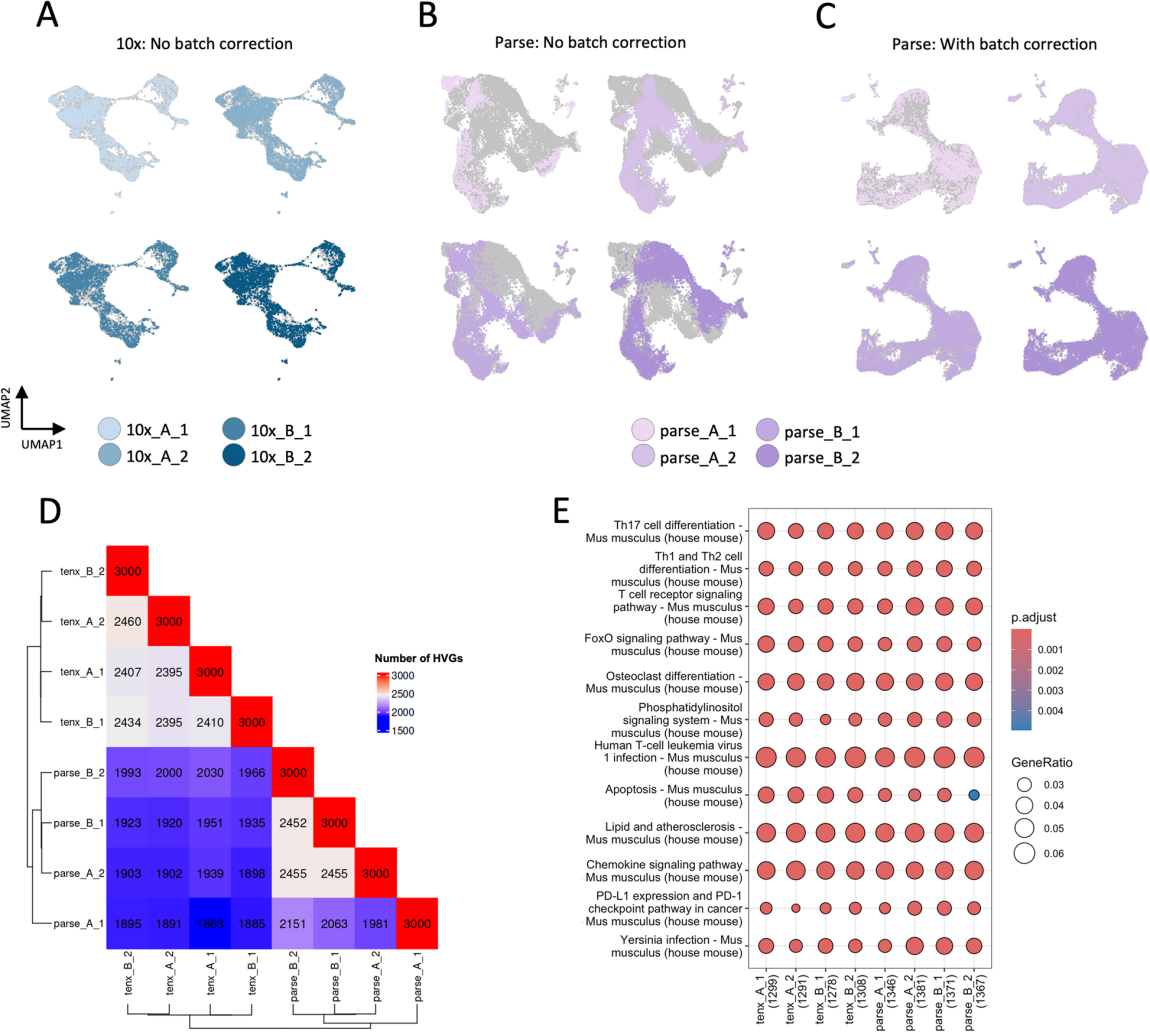
Batch effect exploration. (**A**) UMAP representation of 10x data without batch effect correction, (**B**) UMAP representation of Parse data without batch effect correction, (**C**) UMAP representation of Parse data with batch effect correction, (**D**) Overlap in HVGs between samples, (**E**) KEGG pathways enriched in HVGs for each sample

We next asked whether the batch effects were associated with the significant variability in detected gene and UMI numbers between Parse samples. Such technical effects might hinder downstream analyses, such as cell type annotation or differential expression analysis. We started by performing quality control to exclude the low-quality cells (Figure S1B, C). Next, we performed a standard scRNA-seq analysis workflow that included normalization, dimensionality reduction, and clustering separately on 10x and Parse datasets. We then examined whether the samples were well-aligned on the UMAP plot to assess the presence of batch effects. We first examined the 10x data and observed that cells from biological and technical replicates were well-aligned (Fig. 3A). However, the replicates of the Parse data formed distinct cell communities (Fig. 3B). If the cells of the same type form distinct clusters due to technical variability between the replicates, such artificial clusters could be mistaken for the *bona-fide* biological states. Therefore, we performed a batch effect correction to eliminate the variability between the replicates in Parse data. After the data integration, we observed that Parse replicates were well-aligned (Fig. 3C).

We further investigated the differences between the replicates in each dataset. Most scRNA-seq workflows rely on reducing the dimensionality of the data by selecting only highly variable genes (HVG) in each sample. We performed this procedure and compared the selected genes between replicates and technologies. The number of common HVGs between the assays was lower than the overlap within each assay (Fig. 3D). The pairwise HVG overlaps were similar between the 10x samples. In contrast, one sample in Parse data had fewer HVGs in common with other samples (parse_A_1). We then performed KEGG enrichment analysis on the HVGs from each sample. The gene ontology analysis indicated a similar representation of the identified pathways as 10x and Parse samples were enriched for the same biological processes (Fig. 3E). The only difference was in the process "Apoptosis", which had a slightly smaller score in the Parse samples compared to 10x.

### 10x resolves thymocyte populations better than Parse

**Fig. 4:**
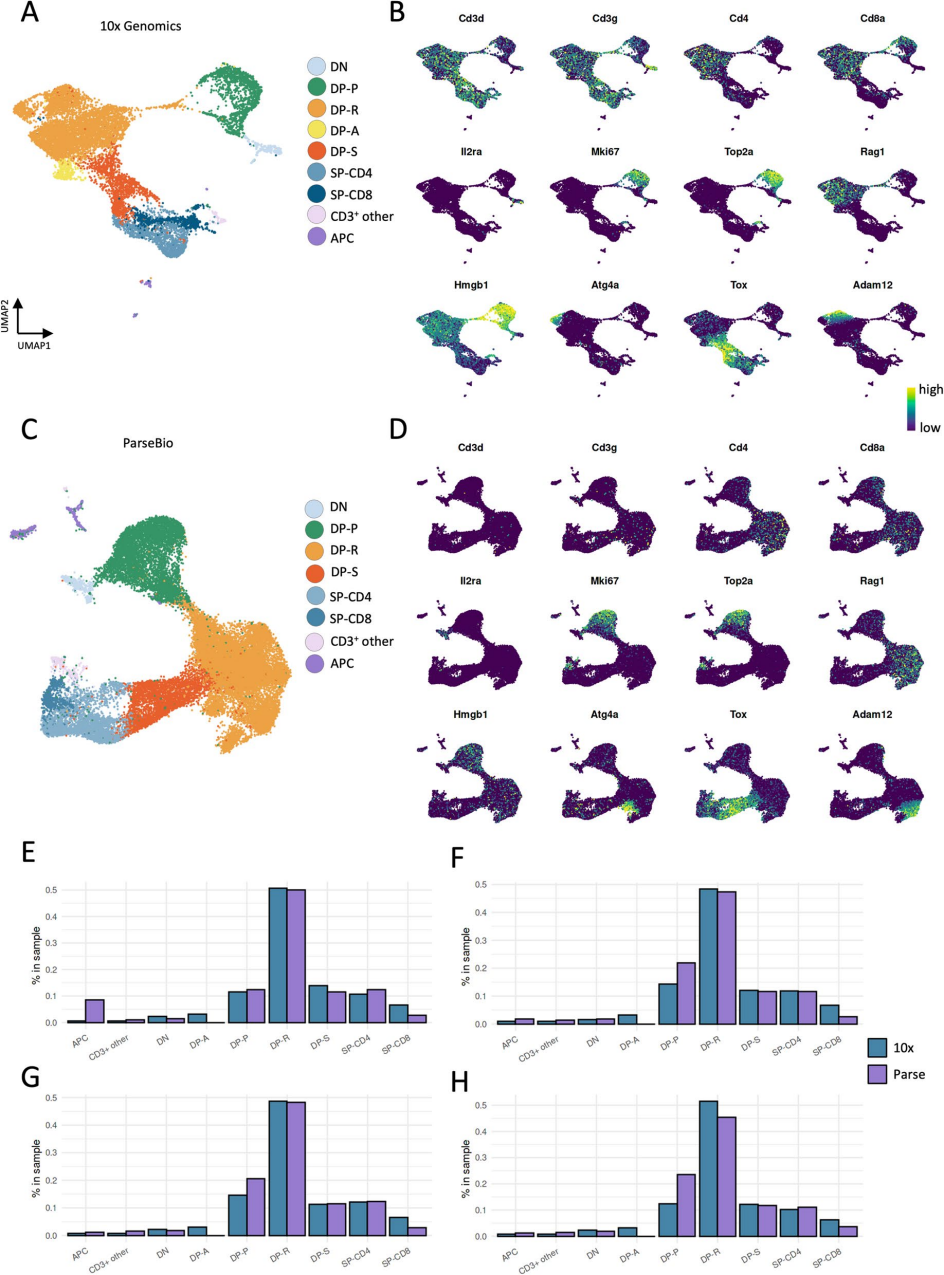
Cell type annotation. UMAP (**A**) and marker genes (**B**) of 10 × data, UMAP (**C**) and marker genes (**D**) of Parse data, Proportion of cells in 10x (blue) and Parse (purple) in replicates (**E**) A_1, (**F**) A_2, (**G**) B_1, and (**H**) B_2

Single-cell datasets often contain diverse cell types and their subsets, and granular annotation of cell types and states is a critical step of scRNA-seq analysis. We, therefore, proceeded to annotate our unsupervised clustering results separately on 10x and Parse data to assess their suitability to decipher the stages of thymocyte differentiation (Fig. 4A-D). We started by examining the *Cd3d* and *Cd3g* expression levels in the unsupervised clustering of 10x and Parse data and observed a pronounced difference in these T cell marker genes between the two datasets. In the 10x dataset, *Cd3d* and *Cd3g* were highly and consistently expressed in almost all clusters (Figure S2A-D). In contrast, the *Cd3d* expression was undetectable in all clusters of the Parse dataset (Figure S3A-D), while its paralogue gene *Cd3g* was present in fewer clusters and at a lower level than in 10x data.

We then grouped the clusters according to the stages of thymic differentiation based on the expression of *Cd4* and *Cd8a* genes. These are represented by double-negative (DN; *Cd4*^*−*^*Cd8*^*−*^), double-positive (DP; *Cd4*^+^*Cd8*^+^), and single-positive (SP; *Cd4*^+^*Cd8*^*−*^ or *Cd4*^*−*^*Cd8*^+^) cell populations. In the 10x data, we identified a *Cd3d*^+^*Cd4*^*−*^*Cd8a*^*−*^ cluster that could be annotated as DN thymocytes. This cluster had a high *Il2ra* expression, a marker of DN cells. Next, we analyzed the Parse data and found that major thymocyte populations were congruent with the 10x data.

We then annotated all *Cd3d*^+^*Cd4*^+^*Cd8a*^+^ clusters in the 10x data. The DP group was subdivided into proliferating (DP-P) and recombining (DP-R) populations based on the expression of genes associated with the cell cycle (*Mki67, Top2a*) and V(D)J recombination (*Rag1*), respectively. The third stage in the differentiation was a DP undergoing selection cluster (DP-S) representing thymocytes in the post-recombination positive selection stage (*Tox*). In addition, we observed a DP apoptotic cluster (DP-A) that was enriched in mitochondrial gene expression (Figure S2C). We used the same markers to annotate DP-P, DP-R, and DP-S thymocyte subsets in the Parse data. However, we did not observe a DP-A cluster in the Parse dataset, as there were no cell communities with a pronounced increase in mitochondrial gene expression. The SP clusters represent the end stage of thymocyte development and have mutually exclusive *Cd4* and *Cd8a* expression patterns. In 10x data, we identified the *Cd3d*^+^*Cd4*^+^*Cd8*^*−*^ SP-CD4 and *Cd3d*^+^*Cd4*^*−*^*Cd8a*^+^ SP-CD8 populations, and these were also distinguishable in the Parse data. The remaining clusters were identified as antigen-presenting cells (APC) and other CD3^+^ cells in 10x data based on distinct *Cd3d* expression level and MHC-II related genes. However, we could not identify these clusters in Parse solely by *Cd3*-related gene expression. We annotated the APC cluster in Parse by expression of *Cd4*, *Cd8a,* and MHC-II related genes but other CD3^+^ cells could not be annotated due to a lack of distinct *Cd3d* or *Cd3g* gene expression in the clusters.

We wondered whether we could leverage the 10x data annotations to identify the similar clusters and proportions in Parse data. For this, we trained a classifier on 10x gene expression values and annotations and obtained predictions for the Parse data. Based on this model, we could accurately annotate the minor thymocyte population, other CD3^+^, in Parse data (Figure S4). However, the model could not identify the DP-A cluster. Interestingly, the model could not distinguish between DP-P and the SP-cell populations.

Next, we studied the proportion of the thymocyte cell types identified using each technology. The DP-R, DP-S, and SP-CD4 proportions were relatively consistent between the technologies and replicates (Fig. 4E-H). However, we noticed significantly fewer SP-CD8 cells in the Parse data across all replicates compared to the 10x data. At the same time, Parse had more DP-P cells than 10x in all replicates. Taken together, these results reveal differences in cell subpopulation detection between 10x and Parse kits.

We further checked if the inconsistent levels of *Cd3d* and *Cd3g* genes in Parse data was because of background noise due to barcode swapping. We used SoupX to remove the background RNA from Parse data but did not observe any significant improvements (Figure S5A-D). The *Cd3d* and *Cd3g* gene expression patterns remained similar, as did the lack of DP-A cells, in the Parse data.

We then performed a validation experiment for Parse data to analyze whether the issue with *Cd3d* and *Cd3g* gene expression levels persists. To this end, we used the Parse WT Mini kit to capture up to 20,000 cells. As in the main experiment, we isolated thymus from two identical young female C57BL/6N mice that were split into two technical replicates each. We performed a similar data analysis workflow, with doublet removal and batch effect correction, and observed that *Cd3-*related gene expression was present poorly in all the clusters, which obstructed the identification of APC and other CD3^+^ cell subsets (Figure S6A-D). In addition, we observed that the SP-CD8 cells could not be distinguished in the Parse mini data based on mutually exclusive *Cd4* and *Cd8a* expression patterns (Figure S6B). We then used the cell classifier trained on 10x data to predict annotations for the Parse mini dataset. The cell classifier identified the APC and other CD3^+^ cells but could not differentiate between DP-P and the SP-cell populations as previously (Figure S7). Furthermore, we performed background RNA removal and no improvement was seen in the issues faced (Figure S8A-D).

### RNA velocity analysis shows agreement between 10x and Parse datasets

The thymocytes undergo constant turnover, marked by the continuous influx of progenitor cells, their specification or apoptosis, and exit from the thymus. Therefore, our datasets presented an excellent opportunity to benchmark computational tools for studying developmental dynamics. Using 10x (10x_A_1, 10x_A_2, 10x_B_1, and 10x_B_2) and Parse (parse_A_1, parse_A_2, parse_B_1, parse_B_2) datasets, we performed the RNA velocity analysis based on the ratio of spliced and unspliced transcripts and examined whether both assays captured this information. The abundance of spliced and unspliced transcripts in 10x were 58% and 39%, respectively, while 3% were classified as ambiguous (Figure S9A). This ratio was inversed in Parse data, where 47% of transcripts were spliced and 53% were unspliced (Figure S9C). We studied whether the velocity vector fields and latent time reflected the thymocyte developmental trajectories. We found that the DP-R cells had the highest latent time values in 10x and Parse (Figure S9B, D). The velocity vectors pointed towards the DP cells and away from both SP and DP-P populations.

## Discussion

The scRNA-seq allows to distinguish transcriptionally different cell populations within complex multicellular tissues. Here, we evaluated the applicability of 10x and Parse scRNA-seq kits to study the dynamics of T cell differentiation in mouse thymus and focused on cell type annotation as a central task in scRNA-seq data analysis.

Our study reports the differences between 10x and Parse kits, as summarized in Table 2, providing a reference for selecting the optimal approach for single-cell analysis. Considering the set-up costs, the Parse protocol does not require the purchase of the microfluidic device (Chromium Controller) but involves more hands-on steps and reagents compared to 10x. However, 10x requires additional reagents and steps for sample multiplexing, which are integrated into the Parse workflow. Technical issues with 10x, such as wetting failures and clogging, are associated with generating gel beds-in-emulsion in the Chromium Controller and can be avoided when using Parse.

**Table 2:** Summary

	Parse	10x
Inter-sample variability	High	Negligible
Intra-sample variability	High	Negligible
Sensitivity in gene detection	Higher	Lower
Minimum number of cells to load	100,000	800
Minimum number of recovered cells	200	500
Pause points	7	7
Benchwork	~ 11 h	~ 7.5 h
Maximum number of samples that can be analyzed in one kit with multiplexing	40 samples with 2500 cells/ sample (1 WT kit)	16 samples with 2500 cells/ sample (1 kit with 4 reactions)
Cell type annotation	Difficult	Easier
Doublet detection	Based on genetic diversity or computational	With hashtags

**Fig. 5:**
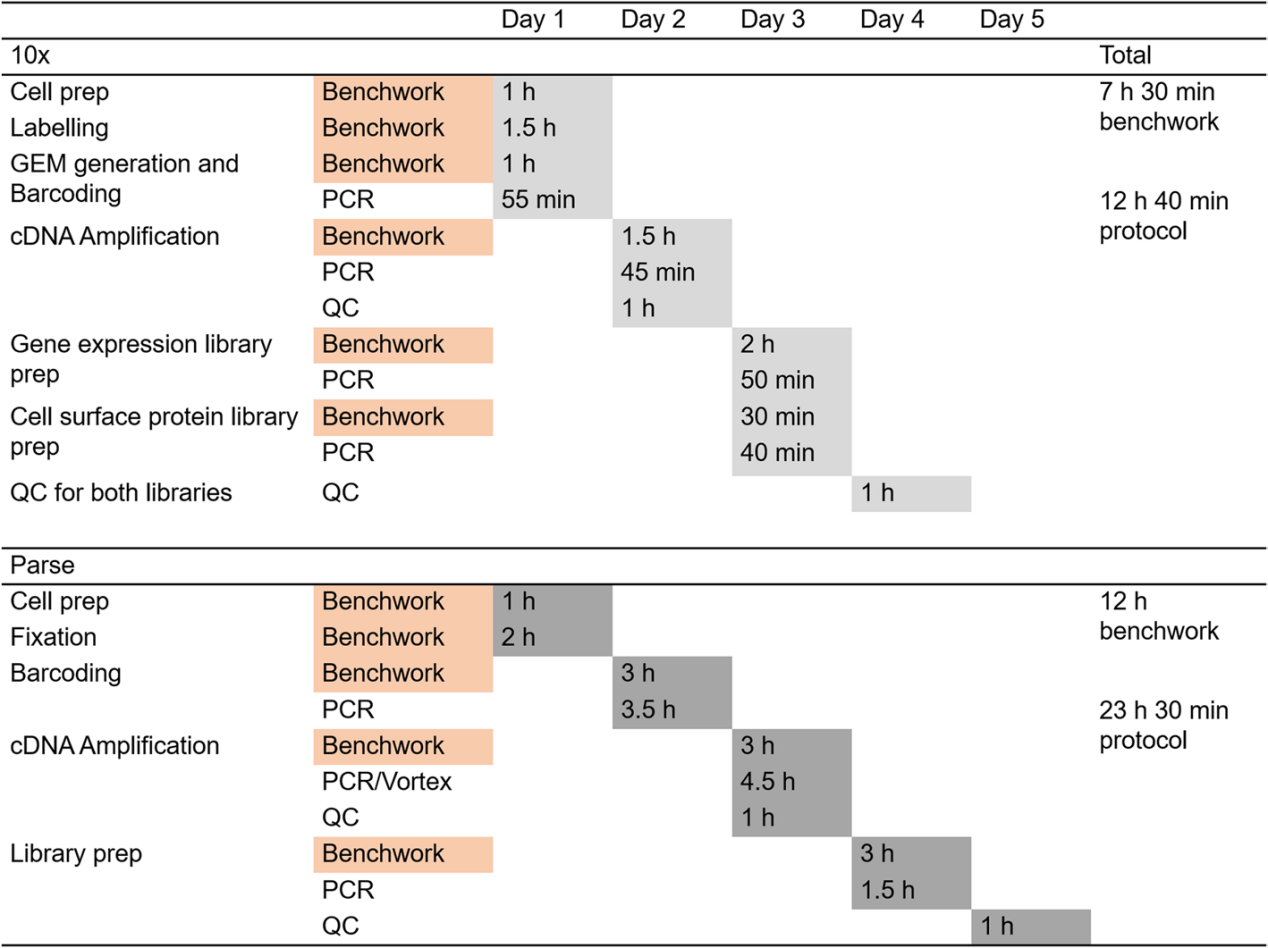
Workflow and time frame required for each kit. Key differences between the steps and time spent on 10x and Parse protocols have been highlighted

The time required for the Parse kit workflow, including sample processing and library preparation, is nearly twice as long as that for the 10x kits with more extensive benchwork (Fig. 5). Both kits have the same pause points, but 10x offers a more flexible three-day workflow, while Parse requires a minimum of a four-day schedule, with one day having more than six hours of workflow without any pause points. Since the cells can be fixed and stored for the Parse kit, it allows samples to be processed from different time points. Although 10x has introduced kits in which cells can be fixed and stored, these require the purchase of a different instrument.

Regarding cell sample loading, both kits require single-cell suspensions with high viability. Parse needs at least 100,000 cells per sample as starting material, whereas the minimum number of cells in 10x is just 800 cells per sample. We obtained slightly higher cell recovery in 10x compared to Parse. Interestingly, three out of four samples in Parse had almost double the expected cell recovery rate. However, this was not replicated in the Parse mini kit, which had an average cell recovery rate of 36.5%, similar to previously reported (16). On the other hand, 10x has consistently shown higher cell recovery rates [16, 34]. Thus, 10x is advantageous over Parse in exploring minor populations such as thymic epithelial cells, which are present in low numbers in the thymus.

Quality metrics of cells in scRNA-seq datasets are important for downstream analysis. Some indicators used to distinguish high-quality cells include the percentage of mitochondrial-coding genes, the presence of doublets or multiplets, and the number of UMIs and genes detected [35]. Our data showed a slightly higher percentage of mitochondrial-coding genes in Parse compared to 10x. Conversely, 10x has been shown to detect a higher proportion of ribosomal-coding genes than other platforms [36]. Similarly, our data showed more than a tenfold increase in the percentage of ribosomal-coding genes and double the percentage of lncRNA-coding genes in 10x compared to Parse. Although 10x has higher cell recovery rates and lower percentage of mitochondrial-coding genes, Parse had lower percentages of ribosomal and lncRNA transcripts. This aspect has been linked to the loss of ambient RNA due to the multiple washing steps in the Parse protocol [21]. The proportion of different gene biotypes in the data impacts the downstream analysis and should be considered according to applications.

Doublets or multiplets are aggregates of two or more cells that are captured into a single droplet, resulting in hybrid transcriptomics. Including these in downstream analysis can lead to false detection of cell types, intermediate cell states, or transcriptomic signatures [37]. In our dataset, we used cell hashing for the 10x samples and were able to distinguish the doublets from the single cells and exclude them from further analysis. Unfortunately, although Parse employs three rounds of barcoding to ensure minimal doublets generated, we found 31% doublets in Parse WT library (4 thymic + 16 non-thymic samples) using *scDblFinder* package as compared to 14% in 10x using hashtags. On the other hand, there were fewer (21%) doublets in Parse mini kit library but we faced issues in identifying the cell types accurately downstream. A previous study benchmarking scRNA-seq kits using mouse and human cells showed a high doublet rate of 49% when using Parse technology [22]. Other studies using human PBMCs and tumor nuclei have shown lower doublet rates in Parse compared to 10x [16, 20]. These results suggest that analyzing different types of tissues or cells in the same Parse kit may aid in better detection of doublets in the libraries generated and improve downstream analysis.

Regarding the UMIs and genes detected, Parse technology identified nearly double the genes detected by 10x at similar sequencing depth. However, the considerable variability in the detected UMIs and genes between the technical replicates in Parse data suggested poor reproducibility compared to 10x. The high batch effect between samples in Parse data may be due to the minute differences in protocol introduced during the fixation, freezing, thawing, or filtration steps. While the integration of computational batch correction tools into the scRNA-seq data workflow complicates the data analysis pipeline, it becomes essential to utilize these tools while analyzing samples from multiple time-points or large number of samples in different runs.

In terms of cluster annotations, we found 10x efficiently differentiated between all major thymocyte populations. Further analysis distinguished between various subsets of DP cell populations as published previously [27]. In contrast, Parse did not show the expected *Cd3d* and *Cd3g* gene expression levels in any of the kits used. This resulted in difficulty in accurately annotating the minor thymocyte populations. We also could not identify the DP-A cluster in Parse. Additionally, Parse mini kit could not separate SP-CD4 and SP-CD8, with most clusters showing *Cd4* expression. While Parse has been claimed to accurately identify rare populations due to its ability to detect genes with low expression levels, this possibly hindered the distinction of SP-CD4 and SP-CD8 [16]. Furthermore, the removal of background RNA from the Parse data did not improve any of the issues mentioned in the respective kits. Overall, 10x is more efficient in clustering the thymocyte subsets. Additionally, we found comparable cell trajectories in the thymic populations between the two methods despite differences in the ratio of spliced and unspliced transcripts.

In conclusion, both methods have advantages and limitations that need to be considered when choosing the appropriate scRNA-seq kit for experiments. 10x can distinguish major subsets in the thymus with fewer cells loaded and a smaller workload but requires specialized instrumentation. Parse is more sensitive to cell populations with lower gene expression levels, which can be a limitation and has a longer protocol, but provides the opportunity to run samples from multiple time points together without the instrument.

## Methods

### Animals

Young (2-month-old) female C57BL/6N mice were used for the experiments. This study was conducted in accordance to the permission from the Ministry of Regional Affairs and Agriculture (Estonia) and approved by the Animal Experiments Ethics Committee at the Ministry (Protocol No. 224). All methods were performed in accordance with relevant guidelines and regulations. This study was carried out in compliance with the ARRIVE guidelines (Animal Research: Reporting of In Vivo Experiments). Young mice were acquired from Laboratory Animal Centre, Institute of Biomedicine and Translational Medicine, Tartu. Mice were housed communally with a standard 12 h dark cycle and fed ad libitum. Mice were euthanized by administering an overdose of ketamine and xylazine intraperitoneally.

### Tissue processing and cell isolation

Briefly, thymi from two female C57BL/6N mice aged two months were isolated, and each lobe was separated and processed separately as a technical replicate. Each thymus lobe was mashed between frosted glass slides and collected in RPMI containing collagenase/dispase and DNase I. The thymic lobe samples were incubated at 37 °C with gentle stirring for 15 min, washed with FACS buffer, filtered through 100 µ filter, and counted using LUNA-FL™ Dual Fluorescence Cell Counter (Logos Biosystems). After counting, each sample was divided equally and further processed by Chromium Next GEM Single Cell 3' Reagent Kit v3.1 (10x Genomics; 10x_A1, 10x_A2, 10x_B1, 10x_B2) and Parse Evercode Whole Transcriptome (WT) kit v2 (Parse Biosciences; parse_A1, parse_A2, parse_B1, parse_B2), respectively. The four 10x samples were multiplexed using TotalSeq™-B hashtag antibodies (BioLegend), washed, counted, and 20,680 cells were loaded in a single reaction using the 10 × 3' kit as per the manufacturer's protocol. The Parse samples were fixed using the Fixation Kit (Parse Biosciences), counted, and stored at -80 °C as per manufacturer's protocol. We loaded 19,230 cells per sample for a total of 384,600 cells from 20 samples (4 samples for the benchmarking experiment and remaining 16 unrelated samples to utilize the maximum capacity of the kit). The samples were multiplexed and processed using the Parse WT kit as per manufacturer's protocol. In addition, we repeated the experiment with the same experimental set up using Parse Mini v2 kit (Parse Biosciences) to confirm our findings from the WT kit. All libraries were sequenced as paired-end 150-bp reads using the Illumina NovaSeq 6000 platform.

### Raw data processing and quality control

The 10x data FASTQ files were mapped to the *mm10* mouse reference (downloaded from the 10x Genomics website) using *CellRanger* software. For Parse data, we first generated an indexed genome using the *mm10* mouse reference downloaded from Ensembl. Next, we used the *split-pipe* tool from Parse to process files from each sub-library. The results from each sublibrary were then combined into one gene expression matrix. The gene expression matrices from 10x and Parse were loaded using the *Seurat* R package [38]. Quality control was conducted on each dataset to remove low-quality and contaminating cells. Outlier cells were excluded based on the distribution of UMIs and the percentage of mitochondrial gene expression. We filtered out the cells with fewer than 500 and over 25,000 UMIs (Figures S1B,C). For 10x, we excluded cells with more than 10% mitochondrial gene expression, while the cutoff was set to 15% for Parse data (Figures S1B,C).

### Doublet detection

The 10x data was demultiplexed based on hashtag read counts using the *HTODemux* function. We used the *scDblFinder* package for the Parse data, which performs computational doublet detection based on gene expression profiles [33]. In summary, *scDblFinder* creates artificial doublet profiles and identifies cells as doublets if their gene expression closely matches the simulated doublets. As the algorithm's results depend on random initialization, we performed ten runs with different random seeds. We then marked a cell as a doublet if it has been called a doublet in more than half of *scDblFinder* runs. This procedure ensures that the number of false-positive doublet calls is reduced.

### Statistical tests

We used the Wilcoxon Rank Sum test to test the differences in the number of UMIs and genes between samples in Fig. 2.

### Clustering and dimensionality reduction

The combined data from each assay (including all samples) were processed using the standard Seurat workflow to investigate the presence of a batch effect. In brief, the data were first normalized using the *SCTransform* method as implemented in the *glmGamPoi* R package [39]. Next, the principal component analysis (PCA), nearest-neighbour graph construction, and UMAP dimensionality reduction were performed on the Pearson residuals. The clusters were identified with the shared nearest neighbour (SNN) modularity procedure implemented in *Seurat*.

To account for the batch effect in Parse data, we performed the data integration workflow as previously described [40]. We first normalized the data for each sample using the *SCTransform* normalization and performed PCA. We then selected 3000 integration features and performed the data integration using the reciprocal PCA procedure. Next, we proceeded with clustering and UMAP analysis as described above. To visualize the gene expression values, we obtained the batch-corrected counts with the *PrepSCTFindMarkers* function.

### Cell type label transfer

To perform cell type label transfer from 10x to Parse data, we used the *CellTypist* Python package as previously described [41]. To this end, we first imported the raw count data and 10x cell type annotations using the *Scanpy* package. Next, we performed a feature selection step by training a classifier model and identifying the top model coefficients for each cell type label. These coefficients correspond to the genes most important for a particular cell subset. Next, we trained a classifier model using the union of the genes identified during the feature selection. Finally, we performed the classification on the Parse data with the majority voting enabled.

### RNA velocity analysis

We used the *scVelo* Python package to perform the RNA velocity analysis on both 10x and Parse data. For 10x, we used the *velocyto* package to obtain the spliced and unspliced matrices from the BAM files [42]. For Parse, the same information was obtained from the *split-pipe* outputs using a script available from Parse Biosciences support website (https://support.parsebiosciences.com). Using the splicing information, we performed the dynamical modelling procedure as previously described.

## Supplementary Information

Additional file 1 : Figure S1. (A) Hashtag demultiplexing results for 10x library, (B) QC for 10x Genomics data. The UMI distribution and mitochondrial expression percentage are shown for each sample. The UMI distribution is shown on a logarithmic scale, with red dashed lines marking QC thresholds: 500 UMIs as the minimum for filtering low RNA content and 25,000 UMIs as the upper limit. The 10% cutoff was used to filter out cells with high mitochondrial expression. (C) QC for Parse WT data. The UMI distribution and mito % are shown for each sample with the same QC thresholds as in panel (B). The UMI distribution is also plotted on a logarithmic scale, and the mito % cutoff is set at 15%. The bar plots in (B) and (C) show the percentage of excluded (red) and retained (green) cells for each library. Figure S2. 10x data (A) clustering, (B) marker gene expression, (C) UMAP with technical metrics overlaid, and (D) top DE genes in each cluster. Figure S3. Parse WT data (A) clustering, (B) marker gene expression, (C) UMAP with technical metrics overlaid, and (D) top DE genes in each cluster after doublet removal and batch effect correction. Figure S4. Cell classifier trained on the 10x library annotated data applied to Parse WT library after doublet removal and batch effect correction. Figure S5. Parse WT data after running SoupX (A) clustering, (B) marker gene expression, (C) UMAP with technical metrics overlaid, and (D) top DE genes in each cluster after doublet removal and batch effect correction. Figure S6. Parse mini data (A) clustering, (B) marker gene expression, (C) UMAP with technical metrics overlaid, and (D) top DE genes in each cluster after doublet removal and batch effect correction. Figure S7. Cell classifier trained on the 10x library annotated data applied to Parse mini library after doublet removal and batch effect correction. Figure S8. Parse mini data after running SoupX (A) clustering, (B) marker gene expression, (C) UMAP with technical metrics overlaid, and (D) top DE genes in each cluster after doublet removal and batch effect correction. Figure S9. Detected RNA splicing in (A) 10x and (C) Parse WT data. RNA velocity vectors and pseudotime in (B) 10x and (D) Parse WT data.

## Data Availability

The datasets supporting the conclusions of this article are available in GEO repository, GSE253406.
