# Supplementary material for: Comparative transcriptomic analyses of thymocytes using 10x Genomics and Parse scRNA-seq technologies
Source: BMC Genomics. 2024 Nov 11;25:1069. doi: 10.1186/s12864-024-10976-x (PMC11552371; doi:10.1186/s12864-024-10976-x)
Supplement: Supplementary file 1 — Additional file 1 : Figure S1. (A) Hashtag demultiplexing results for 10x library, (B) QC for 10x Genomics data. The UMI distribution and mitochondrial expression percentage are shown for each sample. The UMI distribution is shown on a logarithmic scale, with red dashed lines marking QC thresholds: 500 UMIs as the minimum for filtering low RNA content and 25,000 UMIs as the upper limit. The 10% cutoff was used to filter out cells with high mitochondrial expression. (C) QC for Parse WT data. The UMI distribution and mito % are shown for each sample with the same QC thresholds as in panel (B). The UMI distribution is also plotted on a logarithmic scale, and the mito % cutoff is set at 15%. The bar plots in (B) and (C) show the percentage of excluded (red) and retained (green) cells for each library. Figure S2. 10x data (A) clustering, (B) marker gene expression, (C) UMAP with technical metrics overlaid, and (D) top DE genes in each cluster. Figure S3. Parse WT data (A) clustering, (B) marker gene expression, (C) UMAP with technical metrics overlaid, and (D) top DE genes in each cluster after doublet removal and batch effect correction. Figure S4. Cell classifier trained on the 10x library annotated data applied to Parse WT library after doublet removal and batch effect correction. Figure S5. Parse WT data after running SoupX (A) clustering, (B) marker gene expression, (C) UMAP with technical metrics overlaid, and (D) top DE genes in each cluster after doublet removal and batch effect correction. Figure S6. Parse mini data (A) clustering, (B) marker gene expression, (C) UMAP with technical metrics overlaid, and (D) top DE genes in each cluster after doublet removal and batch effect correction. Figure S7. Cell classifier trained on the 10x library annotated data applied to Parse mini library after doublet removal and batch effect correction. Figure S8. Parse mini data after running SoupX (A) clustering, (B) marker gene expression, (C) UMAP with technical metrics overlai [file 12864_2024_10976_MOESM1_ESM.pdf]

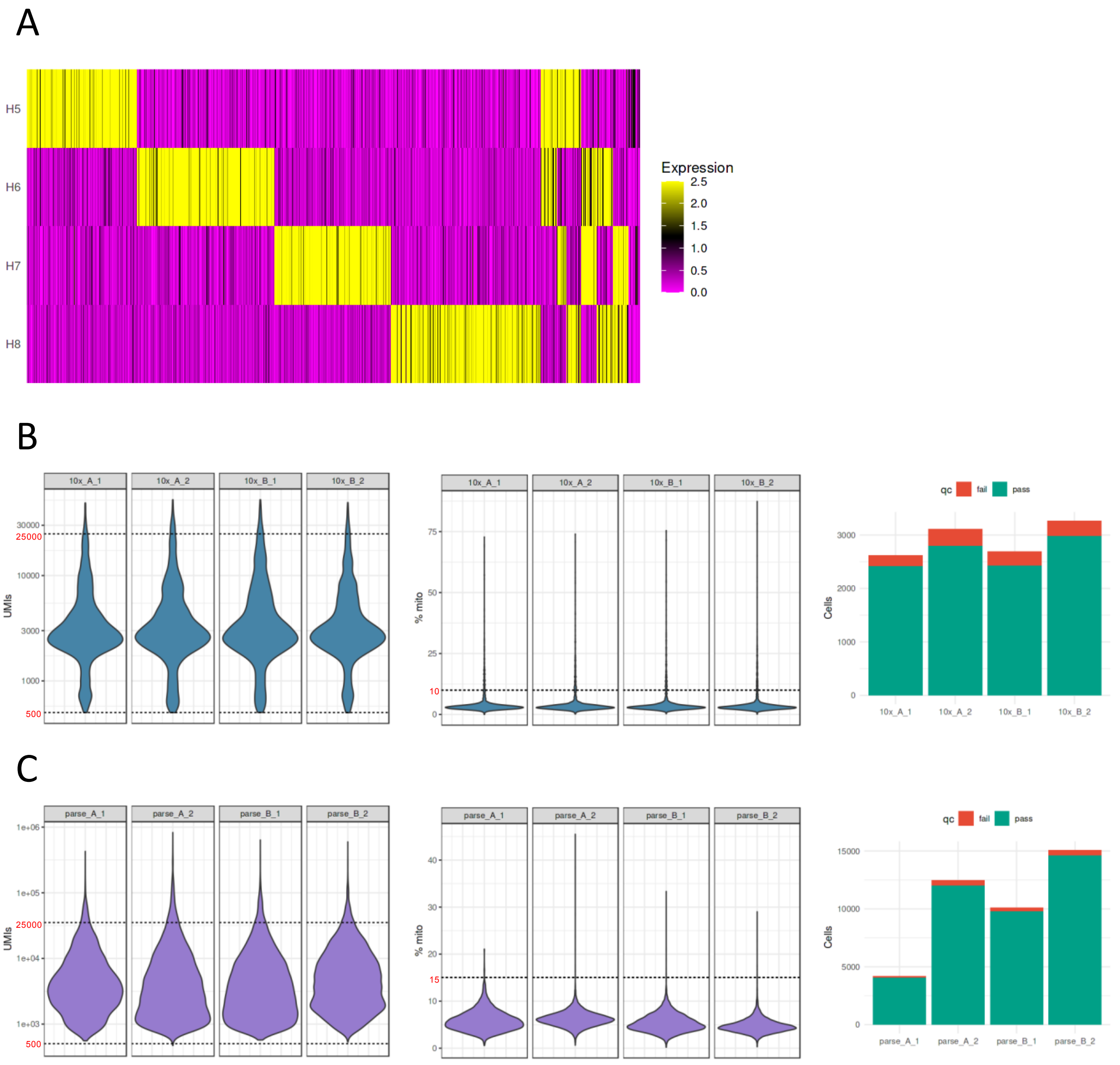

Figure S1. (A) Hashtag demultiplexing results for 10x library, **(B)** QC for 10x Genomics data. The UMI distribution and mitochondrial expression percentage are shown for each sample. The UMI distribution is shown on a logarithmic scale, with red dashed lines marking QC thresholds: 500 UMIs as the minimum for filtering low RNA content and 25,000 UMIs as the upper limit. The 10% cutoff was used to filter out cells with high mitochondrial expression. **(C)** QC for Parse WT data. The UMI distribution and mito % are shown for each sample with the same QC thresholds as in panel (B). The UMI distribution is also plotted on a logarithmic scale, and the mito % cutoff is set at 15%. The bar plots in **(B)** and **(C)** show the percentage of excluded (red) and retained (green) cells for each library

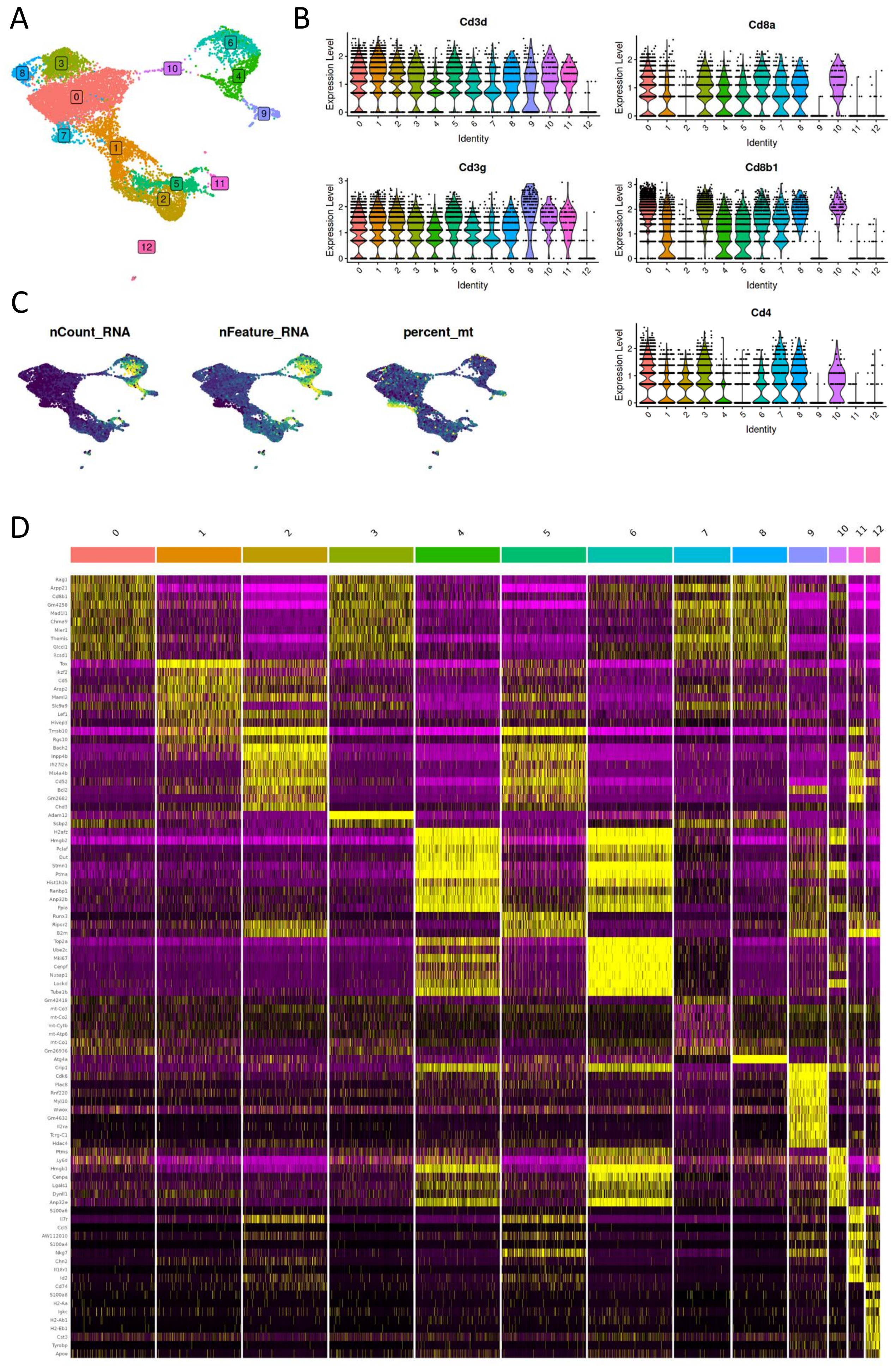

Figure S2. 10x data (A) clustering, (B) marker gene expression, (C) UMAP with technical metrics overlaid, and (D) top DE genes in each cluster

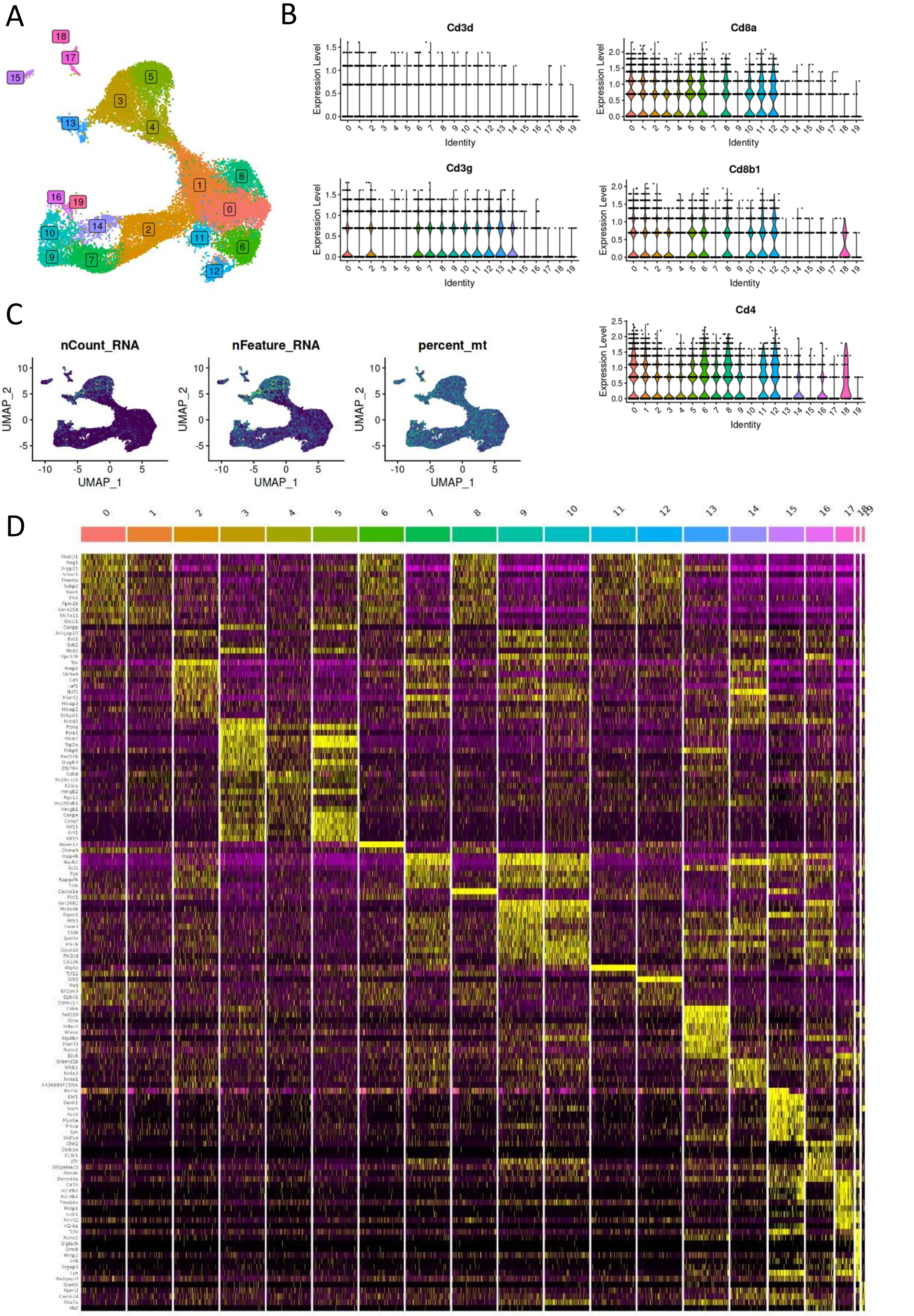

Figure S3. Parse WT data (A) clustering, (B) marker gene expression, (C) UMAP with technical metrics overlaid, and (D) top DE genes in each cluster after doublet removal and batch effect correction

Parse WT library

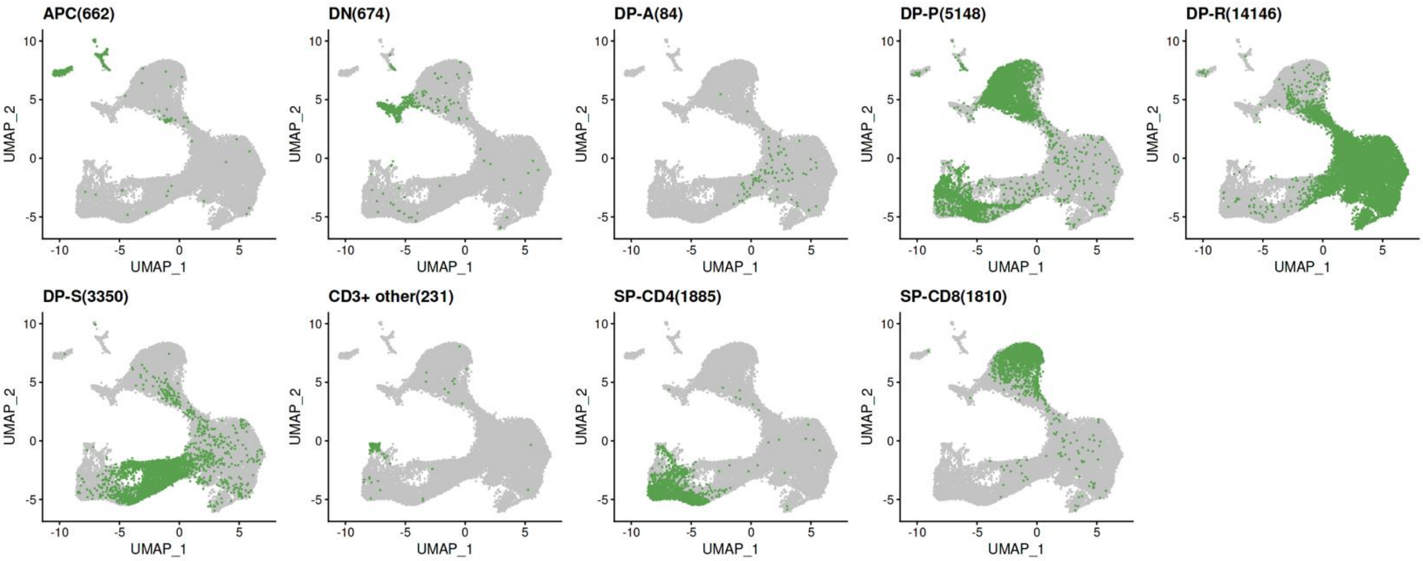

Figure S4. Cell classifier trained on the 10x library annotated data applied to Parse WT library after doublet removal and batch effect correction

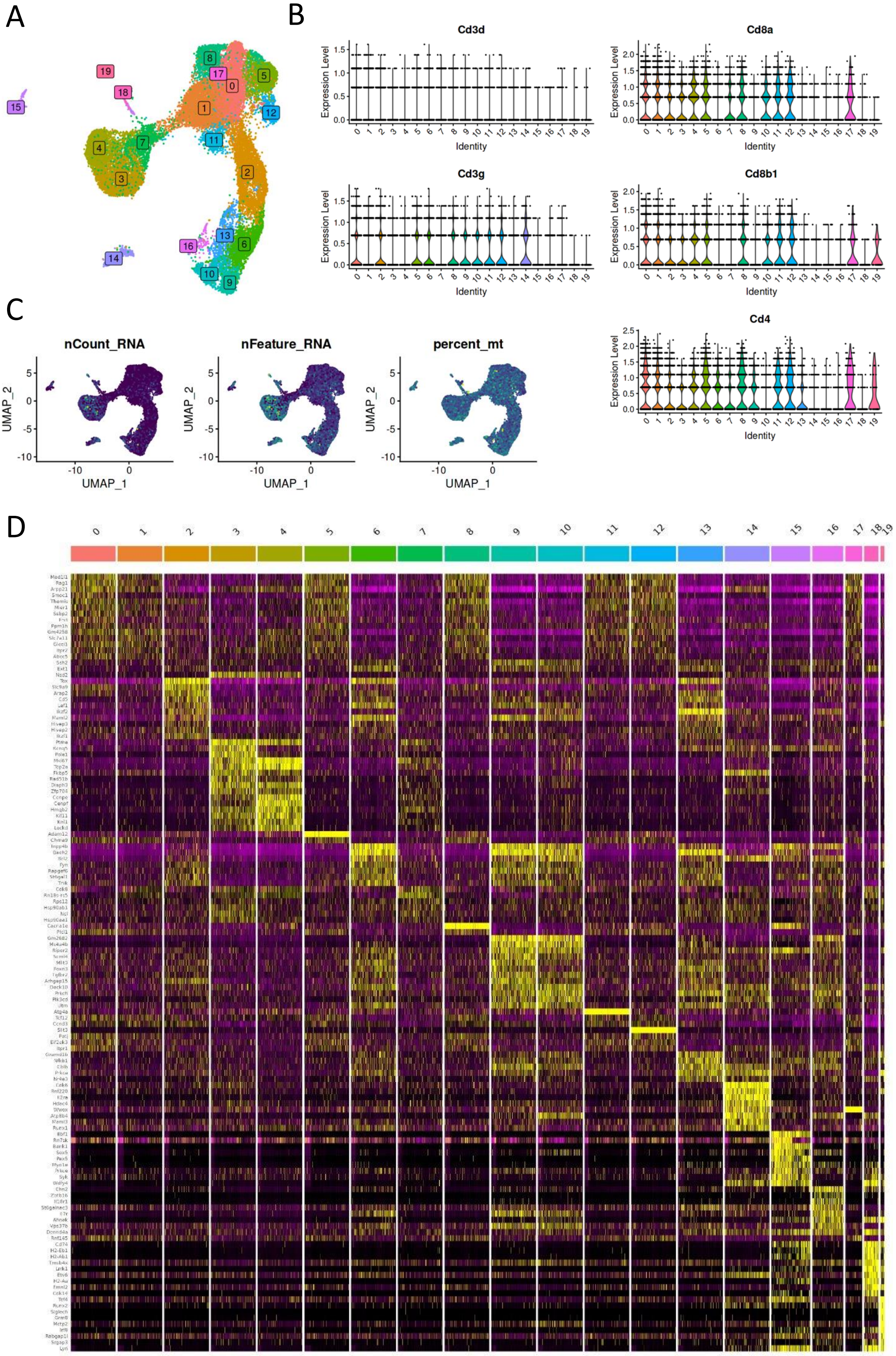

Figure S5. Parse WT data after running SoupX (A) clustering, (B) marker gene expression, (C) UMAP with technical metrics overlaid, and (D) top DE genes in each cluster after doublet removal and batch effect correction

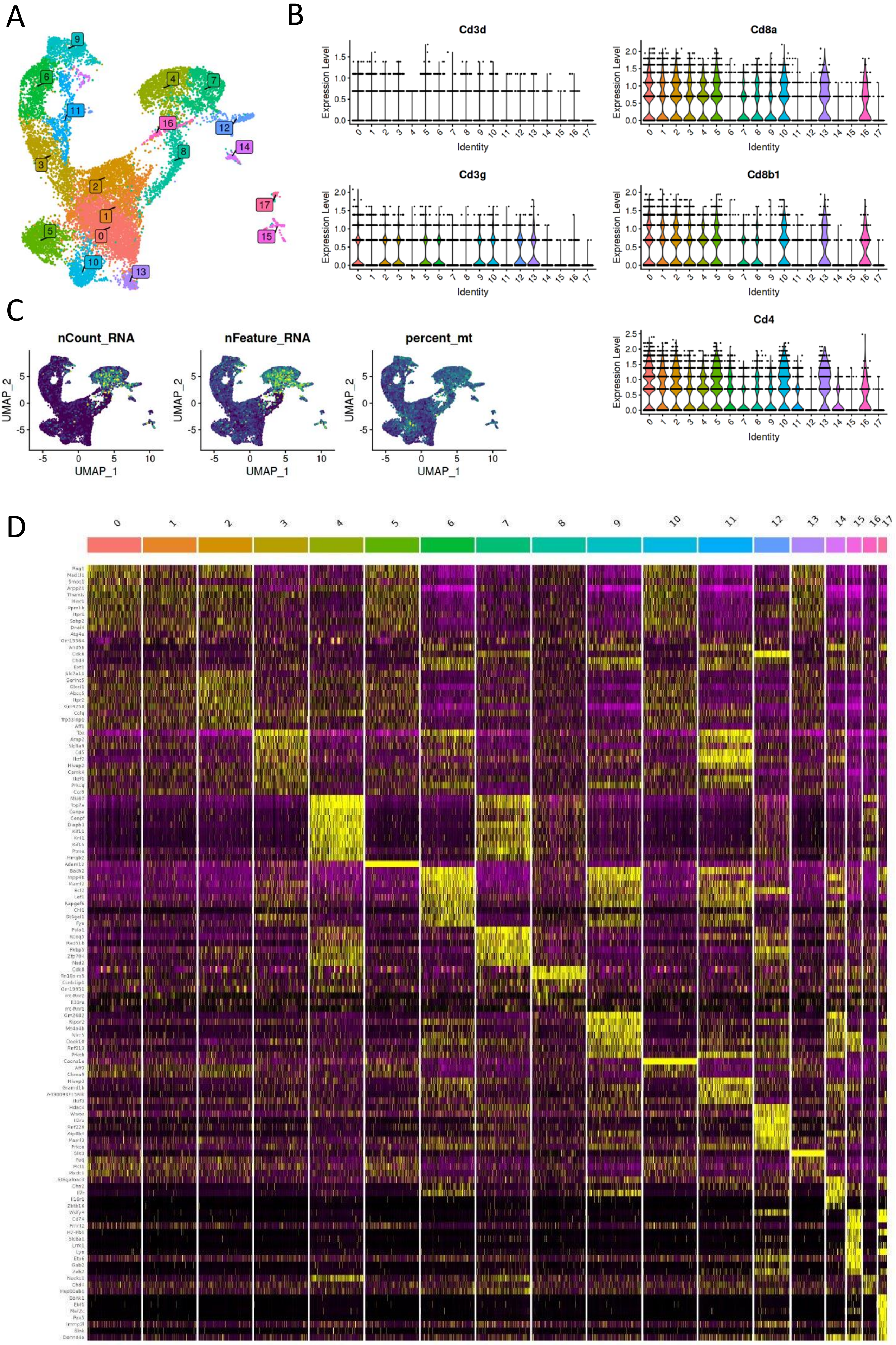

Figure S6. Parse mini data (A) clustering, (B) marker gene expression, (C) UMAP with technical metrics overlaid, and (D) top DE genes in each cluster after doublet removal and batch effect correction

Parse mini library

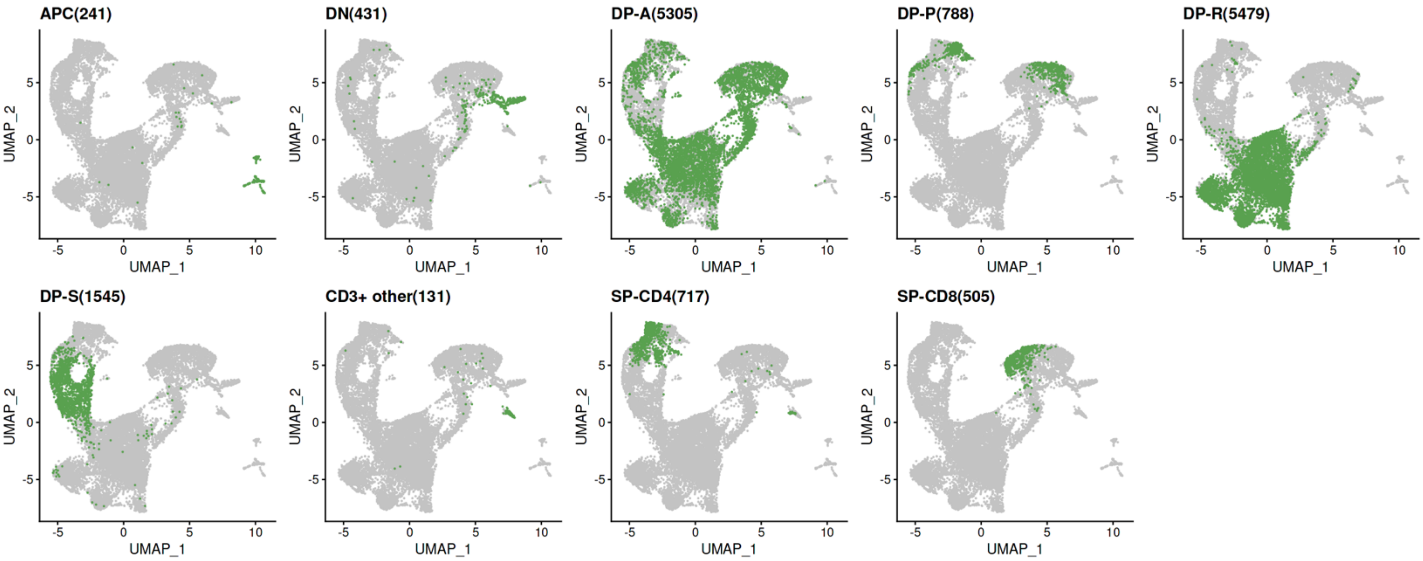

Figure S7. Cell classifier trained on the 10x library annotated data applied to Parse mini library after doublet removal and batch effect correction

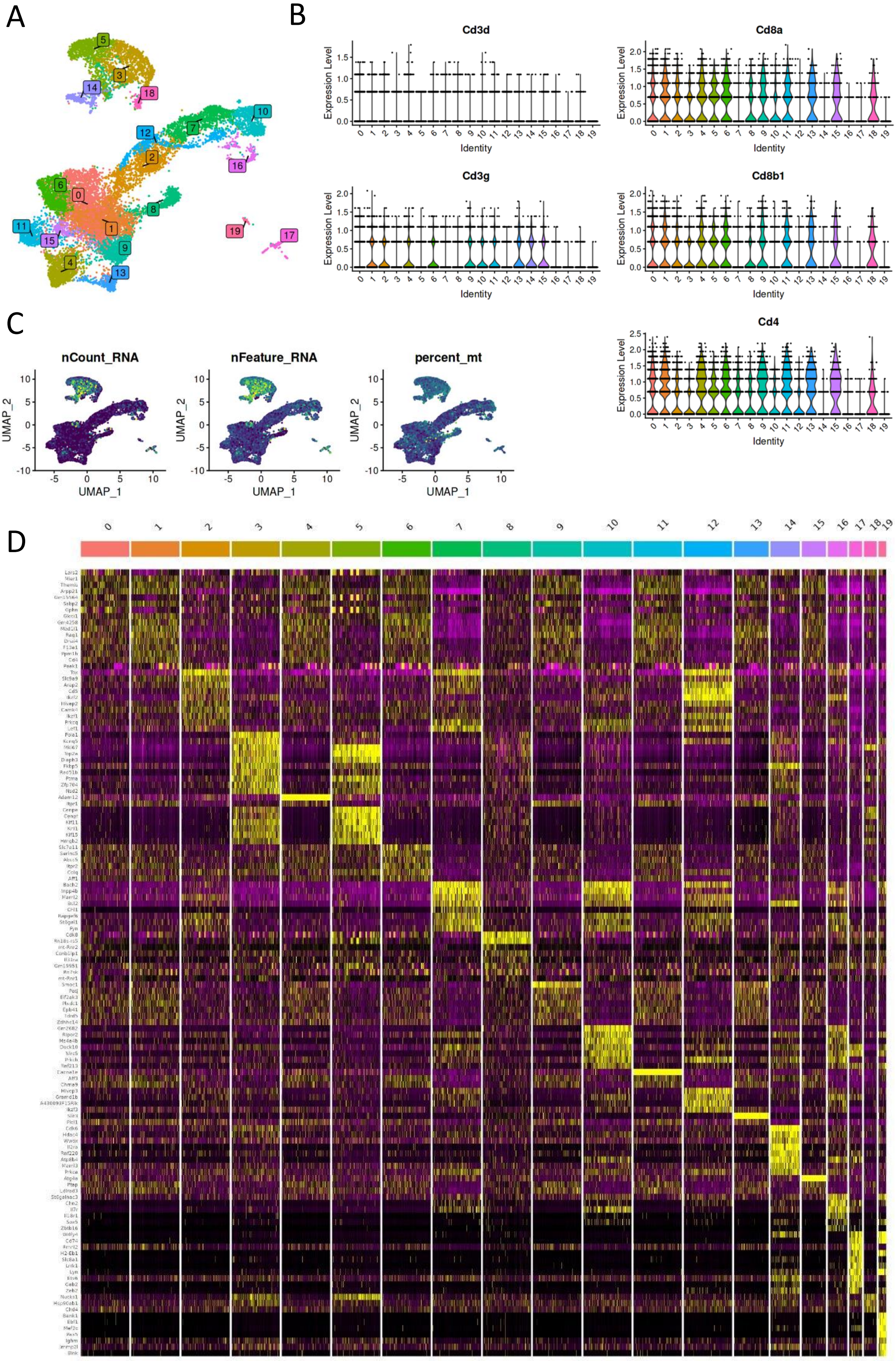

Figure S8. Parse mini data after running SoupX (A) clustering, (B) marker gene expression, (C) UMAP with technical metrics overlaid, and (D) top DE genes in each cluster after doublet removal and batch effect correction

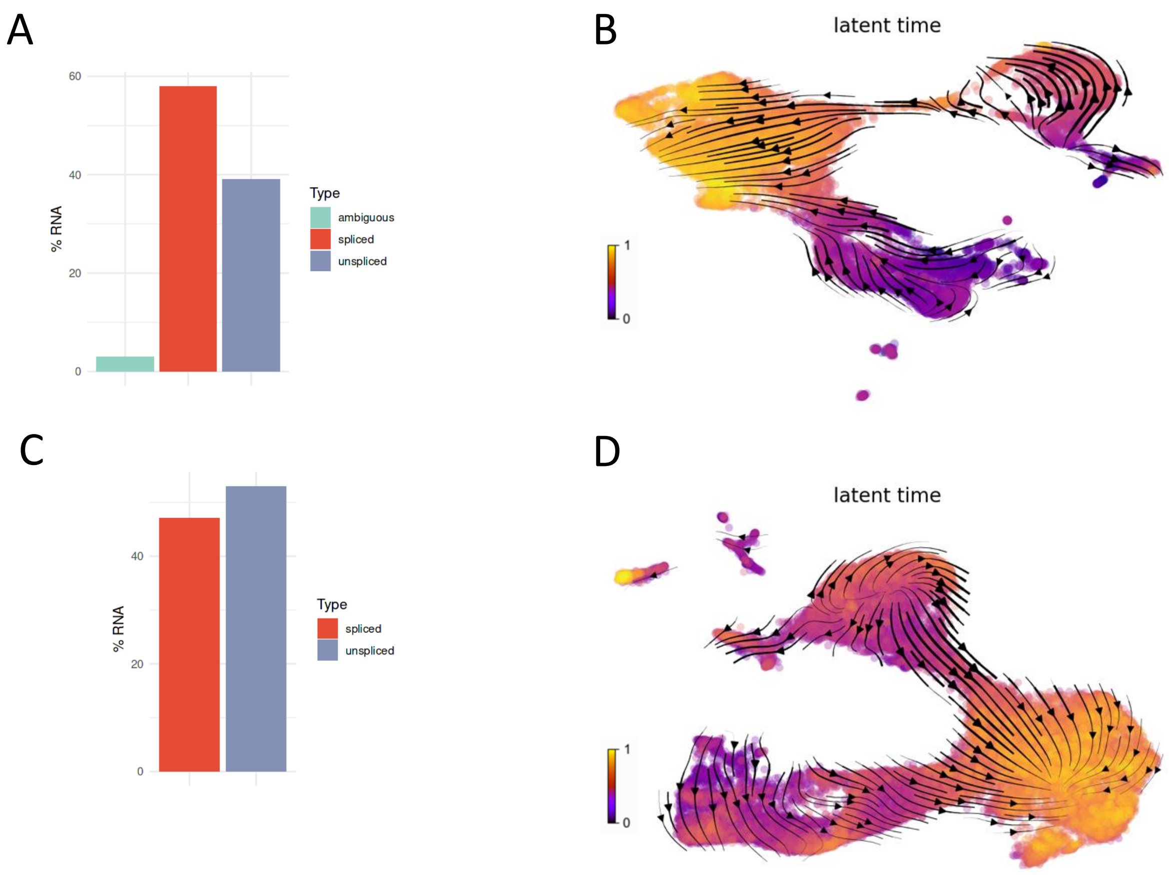

Figure S9. Detected RNA splicing in (A) 10x and (C) Parse WT data. RNA velocity vectors and pseudotime in (B) 10x and (D) Parse WT data
